# Epidemiological investigation of measles outbreak in a refugee settlement in Lamwo District, Uganda

**DOI:** 10.11604/pamj.supp.2025.51.1.47672

**Published:** 2025-07-25

**Authors:** Bob Omoda Amodan, Emmanuel Ssendikwanawa, Josephine Namayanja, Bob Rolex Opio, Godfrey Biroma, Job Morukileng, Denis Ochula, Micheal Baganizi

**Affiliations:** 1 Uganda National Expanded Programme on Immunization, Ministry of Health Headquarters, Kampala, Uganda,; 2 World Alliance for Lung and Intensive Care Medicine, World Alliance for Lung and Intensive Care Medicine in Uganda, Kampala, Uganda,; 3 Health Department, Lamwo District Local Government, Lamwo, Uganda,; 4 Uganda National Institute of Public Health, Uganda Public Health Fellowship Program, 4th Floor, Lourdel towers, Plot 1, Lourdel Road Nakasero, Kampala, Uganda

**Keywords:** Measles, outbreak, elimination, vaccination

## Abstract

On 26^th^ September 2022, the Ministry of Health received a notification of a measles outbreak in Lamwo District. Eight (8) out of the eleven blood samples collected from suspected case-persons of Palabek Gem and Palabek Ogilli Sub-Counties for testing turned positive for measles-specific IgM antibodies at the UVRI. On 29^th^ September 2022, a team of Epidemiologists from the Ministry of Health was deployed. We conducted an epidemiological investigation to determine the scope of the outbreak, and associated risk factors to prevent further transmission. We defined a probable and confirmed case in line with Uganda’s national technical guidelines for integrated disease surveillance and response. We reviewed outpatient and inpatient health facility records of all health facilities to generate a line list from 1^st^ July to 30^th^ September 2022. We then conducted a case-control study, with Mantel Haenszel odds ratios to determine the factors associated with the outbreak. The study identified fifty-six measles cases (48 probable and eight confirmed), with 54% being male, and 89% being South Sudanese nationals. The highest attack rate was among children <1 year (51/10,000) and Zone 8 parish in the Palabek Refugee Settlement (72/10,000). The index case was a 6-year-old South Sudanese boy who entered Uganda on 7^th^ July 2022 and later developed symptoms on 24^th^ July 2022. In this study, we estimated vaccination coverage and the vaccine effectiveness at 63% and 72%, respectively. Malnutrition (ORMH=3.73, 95% CI: 1.22-11.43) and receiving one or no doses of measles vaccine (ORMH=7.0, 95% CI: 2.8-17.6) were the risk factors. We recommended mass administration of vitamin A supplementation and a measles vaccine dose to children under five years of age regardless of the vaccination status. To prevent future outbreaks, we recommended improvement of nutrition and implementation of periodic measles follow-up vaccination campaigns.

## Introduction

Measles spread through respiratory droplets and direct contact with secretions from the nose, mouth, or throat of an infected person. With a reproductive number (R0) of 12-18, measles is thus one of the most contagious diseases [1]. The incubation period for measles varies from 7 to 21 days following exposure to the virus, with fever manifesting within this period. The onset of the characteristic rash typically occurs approximately 14 days after exposure [2,3]. Measles is a leading cause of vaccine-preventable morbidity and mortality, especially in Africa and Asia. Compared to 2021, the global incidence of measles increased by 18%, rising from 7.8 million to 9.2 million cases in 2022. Similarly, there was an increase in deaths from 95,000 in 2021 to 136,200 in 2022. Over 95% of measles deaths occur in countries with low per capita incomes and weak health infrastructures [4].

In the Africa region, the incidence of measles increased by 22%, rising from an estimated 3.6 million cases in 2017 to 4.4 million in 2021. The estimated measles-related mortality fluctuated from 61,166 in 2017 to 104,543 in 2019 before declining to 66,230 in 2021. Notably, Uganda had a measles incidence of 7 per 1,000,000 in 2016, 29 per 1,000,000 in 2017, and peaked at 66 per 1,000,000 in 2018. After a nationwide measles vaccination campaign in 2019, the incidence decreased to 23 per 1,000,000 [5]. Between 2017-2021, measles vaccination efforts averted globally, an estimated 4.5 million measles deaths that could have resulted from outbreaks [6]. Due to the availability of a highly effective and low-cost vaccine and the fact that the disease does not have non-human reservoirs, measles has been targeted for elimination and eventual eradication [7]. WHO through its global framework, set priority targets for measles elimination by 2030 in all the six regions [8]. Whereas the WHO African Region, European Region, South-East Asia, Eastern Mediterranean Region, and Western Pacific Region have not eliminated measles, the WHO Region of the Americas was declared to have successfully eliminated measles in 2024 [9].

Achieving measles elimination requires a comprehensive strategy focused on maintaining strong surveillance systems, addressing vaccine hesitancy, and attaining high vaccination coverages of 95% during routine immunization and supplementary immunization to cover the immunity gaps in the population. Additionally, strengthening healthcare systems and cross-border collaborations help to the stop transmission of measles between countries and regions [8]. Measles outbreaks commonly occur in areas with no measles vaccination or low vaccination coverage especially when the measles virus gets imported [9]. The WHO position paper recommends the use of two doses of measles-containing vaccine (MCV) at 95% coverage [10]. The coverage of measles vaccination across the regions varies significantly, highlighting inequalities in access and utilization of healthcare and immunization services. Globally, the coverage for the first and the second dose of the MCV was 83% and 74%, respectively in 2023, which is below the 95% target needed to achieve herd immunity. In Africa, measles vaccination coverage for MCV1 and MCV2 has shown improvement but remains below the global averages. As of 2023, MCV1 coverage in the region was 70%, while MCV2 was disproportionately lower at 49%. Similarly, in Uganda, measles vaccination coverage for MCV1 and MCV2 has shown progress between 2020 and 2024, with MCV1 coverage reaching 93% and MCV2 dropping to 21% from 49% [11].

**Figure 1:**
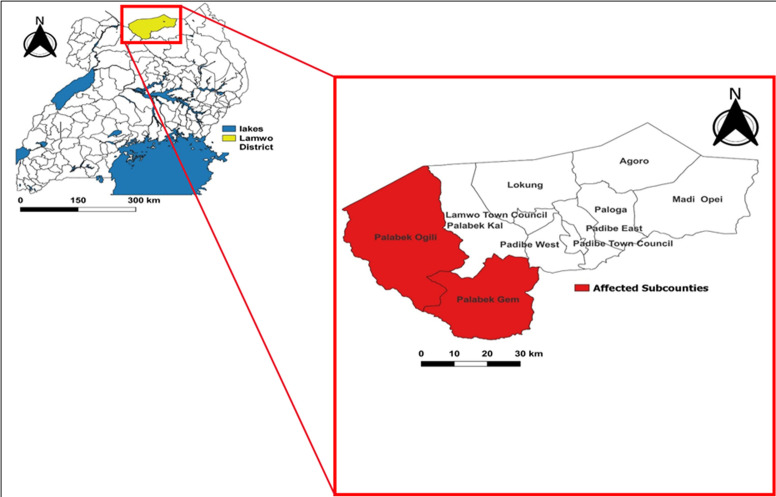
the map showing location of Lamwo District and the sub counties with reported cases

Measles surveillance in Uganda is part of the National Integrated Disease Surveillance and Response System, which requires immediate notification within 24 hours whenever a suspected measles case is identified [12]. When a measles case is suspected, a case investigation form is completed, and then blood samples are collected and submitted to the Uganda Virus Research Institute (UVRI) for testing and Geno sequencing [13]. On 26^th^ September 2022, the Ministry of Health (MoH) received a notification of a measles outbreak in Lamwo District. Eight (8) out of the eleven blood samples collected from suspected case-persons of Palabek Gem and Palabek Ogilli Sub-Counties for testing turned positive for measles-specific IgM antibodies at the UVRI, triggering an immediate investigation (Figure 1). On 29th September 2022, a team of Epidemiologists from the Ministry of Health was led by Bob Omoda Amodan, Field Epidemiologist at UNEPI was deployed to lead the investigation and response to the outbreak. He was assisted by Emmanuel Ssendikwanawa, Epidemiologist from WALIMU, Josephine Namayanja, Field Epidemiologist from UNEPI, Bob Rolex Opio, Medical Officer of Palabek Refugee Settlement, Godfrey Biroma, Data manager from UNEPI, Job Morukileng, Epidemiologist from the Uganda Public Health Fellowship Program, Denis Ochula, District Health Officer of Lamwo, and Micheal Baganizi, UNEPI program Manager, who supervised the whole investigation and response.

Lamwo District, situated in the Northern Region of Uganda, is part of the Acholi sub-region. It borders South Sudan to the north, Kitgum District to the east and southeast, Pader District to the south, Gulu District to the southwest, and Amuru District to the west. The district is about 472 kilometers from Kampala. Spanning an area of 5,595.8 square kilometers, Lamwo District has a population of 213,156 [14]. It is divided into fifteen rural sub-counties and four town councils. The district is also hosts refugees, mainly from South Sudan, within the Palabek Refugee Settlement situated in Palabek Ogili Sub-County. As of June 2023, approximately 79,238 refugees resided in the settlement. The district collaborates with humanitarian organizations like UNHCR to manage the settlement and enhance peaceful co-existence between refugees and local communities. Lamwo District´s healthcare system is supported by thirty-one active health facilities, including 2 Health Centre IVs, 9 Health Centre IIIs, and 20 Health Centre IIs, which all provide immunization services including in the refugee settlement. Three of these facilities are operated by OPM/UNHCR within the Palabek Refugee Settlement, while the rest are managed by government, private organizations, and non-profits. Despite these efforts, challenges in outbreak management, healthcare infrastructure, staffing, and medical supplies continue to affect service delivery in the district. The measles outbreak was first reported in Palabek Gem and Palabek Ogilli Sub-Counties (Figure 1), triggering an immediate investigation.

The administrative MCV1 coverage for Lamwo district was 92%, and the DPT1-MR dropout rate as of FY 2021-2022 was 8.4% (MoH DHIS2 data). This implied that the district immunization performance was at category 1 (High access and High utilization of immunization services). We hypothesized that attending social gatherings, overcrowding, low vitamin A supplementation and poor vaccination status. The objective of this study was therefore to determine the scope of the outbreak, associated risk factors to prevent further transmission.

## Methods

### Case definition

We developed case definitions for probable and confirmed cases. To adopt a probable case definition, the team used the case definitions as provided by the Uganda national IDSR technical guidelines [12]. The team therefore defined a probable case as any resident of Lamwo district presenting with fever and generalized maculopapular rash with one or more of either cough, coryza, or conjunctivitis from 1^st^ July to 30^th^ September 2022. A confirmed case was defined as a probable case with laboratory confirmation (positive IgM antibody test if vaccination status prior to onset of rash was at least 8 weeks) or epidemiological link to confirmed cases in an outbreak in Lamwo district.

### Case finding methods

We reviewed outpatient and inpatient health facility records of all health facilities of Lamwo District to generate a line list of probable and confirmed cases. We further interviewed parents/guardians (if the case was minor) of all line-listed cases to document their detailed clinical history. To find additional cases into the line list, a community visit was conducted, and members of households were asked about the availability of children with measles-like symptoms (snowballing approach). Using a standardized case investigation form, we collected data on the case´s demographics, clinical information, vaccination status, overall health status, nutrition, and other exposure history.

### Descriptive findings

We cleaned our data using Microsoft Excel and later analyzed it using Epi Info 7 software. We assessed the time distribution of the outbreak by constructing epidemic curves from 1^st^ July to 30^th^ September 2022. We further computed measles attack rates by person and place characteristics using National population projections as population denominators [15]. Additionally, dates of onset of measles symptoms were plotted on an epidemic curve to show the trends of the outbreak.

### Hypothesis generation

We conducted hypothesis-generating interviews among randomly selected eighteen (18) measles probable and confirmed cases in Lamwo district. We asked the cases or their caretakers about potential risk factors for measles transmission between 7 and 21 days before symptom onset, including attending social gatherings, attending worship places, visiting health facilities, visiting communal gathering points, vitamin A supplementation in the 6 months before symptom onset, and their vaccination status. Evidence of vaccination included child health cards or, if the health card was missing, parent/guardian´s recall; we probed for details of the site and age at which the child received the measles vaccine. We further asked about exposure factors such as receiving visitors, immunosuppression, malnutrition, and attending large gatherings. All exposure factors that were reported by at least four case-persons were considered potential exposures in the case-control study.

### Analytical study design and rationale

To test the hypotheses that were generated, we conducted a village-matched case-control study in the ten most-affected parishes in Lamwo District. Only probable or confirmed cases were included as cases in the case-control study. A control was defined as any person aged 6 months-10 years without signs and symptoms of measles from 1^st^ July to 30^th^ September 2022 and residing in affected villages of Lamwo.

Cases and controls were included in the study in a ratio of 1:2. Simple random sampling was employed to select controls from the same village as the cases, using village household lists as the sampling frame. We selected only one case in each household. For households with more than one case of measles, the case who developed a rash first was selected for the interview. We recruited and trained sixteen health workers to collect data using the questionnaire. We therefore administered the questionnaire to the case-persons or caregivers, only if the case-persons were found to be minors. We used Epi Info 7 statistical software to analyze the data. We stratified the outcome variable (which was either being a case or control) with selected exposures/risk factors to obtain the Mantel Haenszel odds ratios as measure the level of association. Additionally, we calculated and presented confidence intervals (CIs) for all the selected variables. The chi-square test was used to establish differences between categorical variables and groups. We set a p-value of <0.05 as sufficient evidence to reject the null hypothesis of no difference.

### Vaccination coverage (VC) and vaccine effectiveness (VE)

We estimated the VC using the percentage of controls with a history of measles vaccination in our case-control study, with an assumption that the controls were representative of the general population [16]. We also got the administrative data from the district surveillance officer on the VC of Lamwo District for comparison purposes. We estimated the measles VE using the following formula [17]: VE =1 - OR_MH_, where OR_MH_ is the odds ratio that will be associated with having been vaccinated for at least one dose of measles vaccine from the case-control investigation.

### Laboratory investigations

Blood samples of some suspected measles cases were collected and transported to the Uganda Virus Research Institute (UVRI) for testing and Geno sequencing.

### Environmental investigations

We inspected the health facilities, markets, homes, churches, reception, playgrounds and food distribution centre to observe overcrowding.

## Results

**Figure 2:**
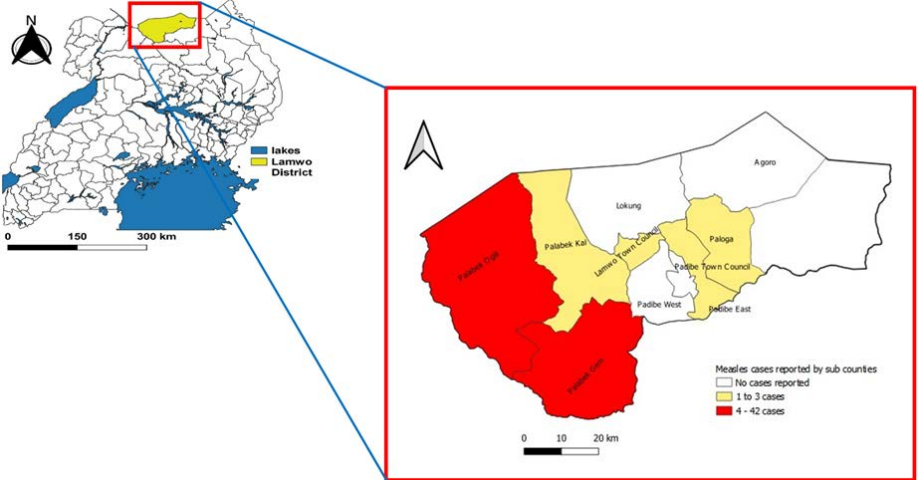
distribution of measles cases by date of rash onset in Lamwo District, Uganda, July to September 2022

Overall, 56 case-persons were identified; 48 of which were categorised as probable and 8 categorised as laboratory confirmed. The epidemic curve showed a propagated measles outbreak (Figure 2) with a steady increase in the number of cases, starting on the 24^th^ July 2022, peaking in between 20th September 2022 and 26^th^ September 2022, and declining by 29th September 2022. The minimum time (incubation period) between the outbreak peaks 13 days. The index case reported an onset of the disease on 24^th^ July 2022 in Olebi Parish, a community that hosts the reception center and neighbouring the refugee settlement. The index case was a 6-year-old South Sudanese boy who entered the Uganda and stayed overnight at the reception centre on 7^th^ July 2022. He (index case) had received measles vaccination and vitamin A supplementation within the last six months preceding the infection but reportedly had poor nutritional status upon arrival from South Sudan. The index case was not laboratory-confirmed. The outbreak was confirmed on 26^th^ September 2022, when 8 out of 11 samples were sent to the EPI laboratory in UVRI, testing positive for measles IgM antibodies.

**Figure 3:**
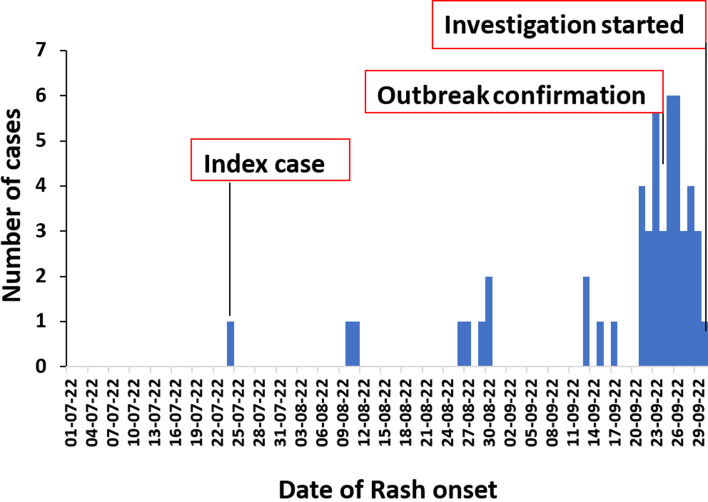
the map showing distribution of cases by the sub counties after outbreak investigation

Out of 56 case-persons, 23 (41%) were in the age group 1-3 years, 30 (54%) were males, 50 (89%) were South Sudanese, and there were no recorded deaths during the outbreak (Table 1). The attack rate was highest (51 per 10,000) in children <1 year of age and slightly higher among males (11 per 10,000) compared to females (9 per 10,000) (Table 1). Of the 56 case-persons, 100% presented with a rash, 56 (100%) case-persons had a fever, 27 (48%) case-persons presented with conjunctivitis; 49 (88%) case-persons had a cough, and 41 (73%) case-persons had coryza. In addition, 5 (9%) case-persons had diarrhoea, 2 (4%) case-persons had pneumonia, and 3 (5%) case-persons had otitis media, all of which were complications of measles. Six (6) out of 19 sub counties were affected by the outbreak, with Palabek Ogili sub county registering the largest number (42) of case-persons and followed by Palabek Gem sub county with 4 case-persons (Figure 3). The overall attack rate was 10 per 10,000, with Zone 8 parish in Palabek refugee settlement being the most affected with 72 per 10,000 (Table 2). Zone 8 parish had 37 (66%) of the case-persons. Alaa, Bungu, Ogilli, Olebi, and Padwat were parishes neighboring the refugee settlements.

**Table 1:** demographic characteristics of measles cases in Lamwo District, Uganda, July to September 2022

Variable	Number of cases (%)	Population at risk	Attack rate/ 10,000
**Age category**			
<1 year	10 (18)	1,977	51
1-3 years	23 (41)	5,605	41
4-5 years	13 (23)	3,516	37
6> years	5 (8.9)	44,123	1.1
**Sex**			
Male	30 (54)	27,155	11
Female	26 (46)	28,067	9
**Nationality**			
Ugandan	5 (8.9)	—	—
South Sudanese	50 (89)	—	—
Congolese	1 (2)	—	—

**Table 2:** attack rate of cases by Parish of residence of measles cases in Lamwo District, Uganda, July to September 2022

Parish	Number of cases (%)	Population at risk	Attack rate (per 10,000)
Alaa	1 (1.8)	2,662	3.8
Bungu	2 (3.6)	3,488	5.7
Ogilli	1 (1.8)	3,829	2.6
Olebi	1 (1.8)	1,947	5.1
Padwat	1 (1.8)	3,249	3.0
Zone 1 (Refugee settlement)	1 (1.8)	3,423	2.9
Zone 4 (Refugee settlement)	6 (11)	8,309	7.2
Zone 5A (Refugee settlement)	5 (8.9)	8,900	5.6
Zone 5B (Refugee settlement)	1 (1.8)	14,279	0.7
Zone 8 (Refugee settlement)	37 (66)	5,136	72
**Total**	**56 (100)**	**55,222**	**10**

### Hypothesis generated

Out of 18 cases selected for exposure hypothesis generation, 14 (78%) had malnutrition during the exposure period, and 12 (67%) stayed in households of size seven and above people. Eleven (61%) case-persons had not received vitamin A supplementation in the 6 months preceding the outbreak. Whereas 13 (72%) case-persons were not vaccinated/or had unknown vaccination status, and 9 (50%) had one dose of measles vaccine. Six (33%) case-persons were South Sudanese nationals who came through the reception centre in the exposure period, and 5 (28%) case-persons were South Sudanese nationals who had gone food distribution centre. Therefore, malnutrition, overcrowded household size, poor vitamin A supplementation, poor measles vaccination status, going through and sleeping at the reception centre, and visiting distribution centre were considered as factors associated with the outbreak.

### Analytical study results

The fifty-six (56) case-persons and 112 control-persons were similar each other by sex (54% of case-persons vs 56% of control-persons were males), and education level of parents/guardians (54% of case-persons vs 53% of controls had not attained formal education). Most (46%) of the case-persons were in the age group of 1-3 years, and contrary, most (63%) the control-persons were in the age group of 4 years and above. Thirty-six (65%) case-persons and 46 (46%) control-persons were malnourished (ORMH=2.6, 95% CI: 1.1-6.0). Fourteen (42%) case-persons and 46 (38%) control-persons had received one or no doses of measles vaccine (ORMH=5.8, 95% CI: 1.4-24.6). Being malnourished (ORMH=3.73, 95% CI: 1.22-11.43) and receiving one or no doses of measles vaccine (ORMH=7.0, 95% CI: 2.8-17.6) were both independently associated with increased odds of measles infection (Table 3). The combination of these two exposures had a reduced effect on the odds of infection (ORMH=2.2, 95% CI: 0.91-5.2) (Table 4).

**Table 3:** factors associated with the measles outbreak in Lamwo District, Uganda, July to September 2022

Risk factor (N=168)	Cases (N=56)	Controls (N=112)	*ORMH (95% CI†)
**n (%)**	**n (%)**
**Nutrition status**			
Well nourished	20 (35)	66 (59)	Ref
Malnourished	36 (65)	46 (46)	2.57 (1.11−5.99)
**Vitamin A supplementation**			
Received	39 (70)	90 (80)	Ref
Did not receive	17 (30)	22 (20)	1.44 (0.47−4.37)
**Measles vaccination**			
Did not receive	23 (41)	42 (37)	Ref
Received	33 (59)	70 (63)	0.28 (0.06−1.25)
**Measles doses ever received (n=103)**			
Two and more doses	19 (58)	13 (19)	Ref
One dose	14 (42)	57 (81)	5.81 (1.37−24.60)
**Going through reception centre**			
No	43 (77)	75 (67)	Ref
Yes	13 (23)	37 (33)	0.56 (0.10−3.28)
**Ever visited distribution centre**			
Visited	46 (82)	105 (94)	Ref
Did not visit	10 (18)	7 (6)	1.63 (0.39−6.75)
**Number occupants at household**			
Less or equal six people	14 (25)	32 (29)	Ref
Seven and more people	42 (75)	80 (71)	0.70 (0.19−2.53)

**Table 4:** common reference group analysis of factors associated with the measles outbreak in Lamwo District, Uganda, July to September 2022

Malnutrition	Zero/ one dose of Measles vaccine	Cases (N=56)	Controls (N=112)	ORMH* (95% CI†)	p-value
		n (%)	n (%)		
**-**	**-**	12 (21)	56 (50)	Ref	
**+**	**-**	8 (14)	10 (9)	3.73 (1.22-11.43)	p<0.05
**-**	**+**	21 (38)	14 (13)	7.0 (2.79-17.56)	p<0.001
**+**	**+**	15 (27)	32 (29)	2.18 (0.91-5.24)	p=0.08

### Vaccine coverage and vaccine effectiveness

The case-control investigation showed that 59% (33/56) of cases compared to 63% (70/112) of controls had a history of measles vaccination (ORMH=0.28, 95% CI: 0.06-1.25). Vaccination coverage was estimated at 59% (95% CI: 56-78%). Using this information, the estimated measles vaccine effectiveness, VE was 1-28% (VE=72%; (95% CI: 0-94%).

## Discussion

This study determined the scope of the measles outbreak and associated risk factors to inform prevention of the further transmission of the epidemic in Lamwo District. Our study found that children under 1 year of age were disproportionately affected, having a high attack rate by the measles outbreak in Lamwo District. Our findings are consistent with previous measles outbreak investigations in Uganda, which similarly reported a higher burden among children aged less than one year [18,19]. This pattern is likely explained by non-adherence of caregivers/parents to take children for measles vaccination as per the schedule in Uganda and South Sudan, which specifies giving the vaccine to children at 9 months of age [20], yet South Sudan had high incidence of measles and therefore considered a high-risk setting. Additionally, there is a high defaulter rate and increased missed opportunities for measles vaccination among children in both Uganda and South Sudan, thus contributing to low vaccination coverage. Several household/community, political, and health system factors such as long distances to vaccination sites, vaccine stockouts, health worker absenteeism, natural hazards, lack of sustained default tracking mechanisms and conflicts, among others also contribute to low vaccine coverage [20].

A considerable proportion of measles cases in this outbreak occurred among South Sudanese nationals, primarily refugees living in settlements within Lamwo District. Notably, Zone 8 parish in a refugee setting alone contributed 66% of all reported cases in the district. In such contexts, conflict, displacement, overcrowding, and limited access to health services often disrupt the timely delivery and uptake of routine vaccinations. Displaced families spend extended periods in transit, navigating insecure and unstable environments where access to immunization is limited or nonexistent. According to UNICEF, about 40% of unvaccinated children in conflict-affected regions miss out on basic immunizations due to breakdown or deliberate destruction of health infrastructure [21]. These challenges collectively create ideal conditions for rapid measles transmission [22].

The investigation also revealed that more male children were affected by measles infection than females. Our findings are in tandem with studies conducted elsewhere in Uganda which reported that males were most affected during a measles outbreak [23]. While measles infection does not inherently discriminate by sex, such disparities may reflect behavioral, social, or cultural dynamics. Boys, for example, may be more likely to engage in outdoor group activities, increasing their chances of contact with infected children. Additionally, gender norms and caregiver perceptions may influence how families seek healthcare or prioritize vaccination for boys versus girls [24]. In some communities, access to and utilization of health services can vary by sex, potentially leading to lower vaccination coverage or delayed treatment for either boys or girls, depending on the local cultural context. A male predominance in measles cases has also been observed in other outbreak investigations, emphasizing the need for gender-sensitive immunization strategies and health education [25].

Furthermore, our investigation revealed that malnourished individuals were approximately four times more likely to develop measles compared to their well-nourished counterparts. The trend is similar to the findings indicated in studies from Ethiopia [26,27] and Sudan [28]. This heightened susceptibility can be attributed to the detrimental effects of malnutrition on the immune system. Specifically, deficiencies in essential nutrients, such as Proteins and Vitamins. Vitamin A deficiency has been associated with increased severity of measles [29]. On the other hand, measles can also exacerbate malnutrition by causing loss of appetite and nutrient malabsorption due to diarrhea. This creates a vicious cycle where malnutrition increases measles severity, and measles, in turn, further depletes nutritional health [30].

Our findings also reported that receiving one dose of measles vaccine increased the odds of measles infection when compared to receiving two or more doses. Indeed, several studies have also indicated that receiving more than one dose of measles vaccine protects children against the infection better than getting only one dose [31-33]. WHO has recommended the use of more than one dose of the vaccine as an important step towards reducing measles-related mortality and morbidity [34].

The vaccine effectiveness in this study is 72% among those vaccinated. This finding is similar to those reported in Uganda [35], Sierra Leone [36] and Ethiopia [37,38]. It is also closely related to the average measles vaccine effectiveness [39]. Vaccine hesitancy is notably lower than findings reported in other parts of Uganda [18,40] and the African region. For instance, in Africa region vaccine effectiveness was 98% in Nigeria, 81% in Egypt, 89% in Gambia, 80% in South Africa, and 84% in Kenya [39]. The disparities in the vaccine effectiveness could be attributed to several factors including nutritional status [41], vaccine storage and handling [42], immune suppression [43-45], interference from maternal antibodies [44,45], vaccine failure [46], and timing [44]. Notably, the major differences in socio-economic conditions and healthcare systems across the countries in the sub-regions cannot be overstated in this study. Vaccine effectiveness can be strategically improved by aiming at a higher coverage with at least two doses of measles vaccine [47].

Limitation: some caregivers or guardians may have struggled to accurately recall the vaccination status of their children or provide vaccination cards for verification. As a result, our findings may have been interfered by recall bias, leading to underestimation or overestimation of vaccination coverage. Additionally, this study has a very small sample size of cases. so, the findings may not be representative of the epidemiological profile in other districts in Uganda or in neighboring countries.

## Conclusion

The study identified 56 measles cases (48 probable and 8 confirmed), with 54% being male, and 89% of the cases were of South Sudanese nationality. The highest attack rate was among children <1 year (51/10,000) and Zone 8 parish in the Palabek Refugee Settlement (72/10,000). The index case was a 6-year-old South Sudanese boy who entered Uganda on 7^th^ July 2022 and later developed symptoms on 24^th^ July 2022. Whereas vaccination coverage was at 63%, the vaccine effectiveness was at 72%. Malnutrition (ORMH=3.73, 95% CI: 1.22-11.43) and receiving one or no doses of measles vaccine (ORMH=7.0, 95% CI: 2.8-17.6) were the risk factors. Therefore, we recommended immediate Vitamin A supplementation and a measles reactive vaccination campaign. To prevent future outbreaks, we recommend implementing periodic measles follow-up supplementary immunization activities, addressing malnutrition, and enhancing measles vaccination at border points and reception centres. Additionally, there is a need to establish regional outbreak coordination centres for sustainable cross-border collaborations.

## References

[1] Guerra FM, Bolotin S, Lim G, Heffernan J, Deeks SL, Li Y, Crowcroft NS (2017). The basic reproduction number (R0) of measles: a systematic review. Lancet Infect Dis.

[2] WHO (2024). Fact sheet for Measles.

[3] Lessler J, Reich NG, Brookmeyer R, Perl TM, Nelson KE, Cummings DA (2009). Incubation periods of acute respiratory viral infections: a systematic review. The Lancet infectious diseases.

[4] Minta AA (2023). Progress toward measles elimination-worldwide, 2000-2022. MMWR Morbidity and Mortality Weekly Report.

[5] Kabami Z, Nakafeero Simbwa B, Namubiru Kizito S, Agaba B, Kayiwa J, Kadobera D (2024). Epidemiological characteristics and trends of measles cases reported through the case-based surveillance system, Uganda, 2016-2020. Uganda Public Health Bulletin Articles.

[6] Masresha BG (2023). Progress toward measles elimination-African Region, 2017-2021. MMWR Morbidity and Mortality Weekly Report.

[7] Moss WJ, Strebel P (2011). Biological Feasibility of Measles Eradication. The Journal of infectious diseases.

[8] World Health Organization (2020). Measles and rubella strategic framework: 2021-2030. Measles and rubella strategic framework: 2021-2030, World Health Organization.

[9] Kumar S, Singh S, Bansal V, Gupta V, Jain R (2024). Unwelcome return: analyzing the recent rise of measles cases in the United States. Proceedings (Baylor University Medical Center).

[10] World Health Organization (2019). Measles vaccines: WHO position paper, April 2017-Recommendations. Vaccine.

[11] World Health Organization (2025). Measles vaccination coverage. Accesed on April.

[12] World Health Organization (2019). Integrated disease surveillance and response technical guidelines, booklet one: introduction section. World Health Organization.

[13] MoH (2012). National technical guidelines for integrated disease surveillance and response.

[14] Uganda Bureau of Statistics National Population and Housing Census 2024 - Final Report-Volume (1). Uganda Bureau of Statistics.

[15] Uganda Bureau of Statistics (2022). Revised Subcounty Population Projections 2015 to 2030 For 146 Districts.

[16] Ma C, Li F, Zheng X, Zhang H, Duan M, Yang Y (2015). Measles vaccine coverage estimates in an outbreak three years after the nation-wide campaign in China: implications for measles elimination, 2013. BMC Infect Dis.

[17] Weinberg GA, Szilagyi PG (2010). Vaccine epidAemiology: efficacy, effectiveness, and the translational research roadmap. The University of Chicago Press.

[18] Namulondo E, Ssemanda I, Komugisha M, Nuwamanya Y, Nsubuga EJ, Wako S (2024). Measles Outbreak in a Refugee Settlement, Kiryandongo District, Uganda, July- October 2023 Research Square.

[19] Kizito SN, Simbwa B, Thomas K, Akuguzibwe R, Sembatya I, Nsubuga E Measles Outbreak Propagated by Visiting a Health Facility. In A refugee Hosting Community, Kiryandongo District, Western Uganda, August 2022.

[20] CORE Group (2018). Immunization in Practice in South Sudan. Juba. South Sudan.

[21] UNICEF Immunization and conflict.

[22] Kouadio IK, Kamigaki T, Oshitani H (2010). Measles outbreaks in displaced populations: a review of transmission, morbidity and mortality associated factors. BMC Int Health Hum Rights.

[23] Nsubuga EJ, Morukileng J, Namayanja J, Kadobera D, Nsubuga F, Kyamwine IB (2022). Measles outbreak in Semuto Subcounty, Nakaseke District, Uganda, June-August 2021. IJID regions.

[24] Ravindran TS, Hebbar P, Bhushan A, Nambiar D, Mishra S, Mohan M Sex and Gender Issues in Health Systems in South Asia: An Overview, Handbook on Sex. Gender and Health: Perspectives from South Asia.

[25] Green MS, Schwartz N, Peer V (2022). Gender differences in measles incidence rates in a multi-year, pooled analysis, based on national data from seven high income countries. BMC Infectious Diseases.

[26] Bukuno S, Asholie A, Girma Z, Haji Y (2023). Measles Outbreak Investigation in Garda Marta District, Southwestern Ethiopia, 2022: Community-Based Case-Control Study. Infection and drug resistance.

[27] Tsegaye G, Gezahegn Y, Tesfaye A, Mulatu G, Bulcha GG, Berhanu N (2022). Measles Outbreak Investigation in Guradamole District of Bale Zone, South Eastern Ethiopia, 2021. Infection and drug resistance.

[28] Kmietowicz Z (2023). Sudan's worsening health conditions see children die of measles and malnutrition. Bmj.

[29] Melenotte C, Brouqui P, Botelho-Nevers E (2012). Severe measles, vitamin A deficiency, and the Roma community in Europe. Emerging infectious diseases.

[30] Belamarich PR (1998). Measles and Malnutrition. Pediatrics In Review.

[31] De Serres G, Sciberras J, Naus M, Boulianne N, Duval B, Rochette L (1999). Protection after Two Doses of Measles Vaccine Is Independent of Interval between Doses. The Journal of infectious diseases.

[32] Control CfD, Prevention The Pink Book: Epidemiology and prevention of vaccine preventable diseases.

[33] Bianchi FP, Mascipinto S, Stefanizzi P, De Nitto S, Germinario C, Tafuri S (2021). Long-term immunogenicity after measles vaccine vs. wild infection: an Italian retrospective cohort study. Human vaccines & immunotherapeutics.

[34] Organization WH (2019). Measles vaccines: WHO position paper, April 2017-Recommendations. Vaccine.

[35] Mupere E, Karamagi C, Zirembuzi G, Grabowsky M, de Swart RL, Nanyunja M, Mayanja H (2006). Measles vaccination effectiveness among children under 5 years of age in Kampala, Uganda. Vaccine.

[36] Sugerman DE, Fall A, Guigui MT, N'Dolie M, Balogun T, Wurie A (2011). Preplanned national measles vaccination campaign at the beginning of a measles outbreak--Sierra Leone, 2009-2010. The Journal of infectious diseases.

[37] Talley L, Salama P (2003). Assessing field vaccine efficacy for measles in famine-affected rural Ethiopia. The American journal of tropical medicine and hygiene.

[38] Desta T, Lemango E, Wayessa D, Wondowossen L, Kerie M, Masresha B (2021). Invalid measles vaccine dose administration and vaccine effectiveness in Ethiopia. Pan African Medical Journal.

[39] Endalamaw D, Nibret E, Munshea A, Mekonnen F, Tadesse S, Zeru T (2024). Measles vaccine effectiveness in African children: a systematic review and meta-analysis. BMC Infect Dis.

[40] Biribawa C, Atuhairwe JA, Bulage L, Okethwangu DO, Kwesiga B, Ario AR (2020). Measles outbreak amplified in a pediatric ward: Lyantonde District, Uganda, August 2017. BMC Infect Dis.

[41] Benn CS, Aaby P, Balé C, Olsen J, Michaelsen KF, George E (1997). Randomised trial of effect of vitamin A supplementation on antibody response to measles vaccine in Guinea-Bissau, West Africa. The Lancet.

[42] Nkwain J, Zambou VM, Nchinjoh SC, Agbor VN, Adidja A, Mbanga C (2025). Deployment of vaccine cold chain equipment in resource-limited settings: lessons from the Gavi Cold Chain Optimization Platform in Cameroon. International Health.

[43] McMorrow ML, Gebremedhin G, van den Heever J, Kezaala R, Harris BN, Nandy R (2009). Measles outbreak in South Africa, 2003-2005. South African medical journal = Suid-Afrikaanse tydskrif vir geneeskunde.

[44] Dabbagh A (2018). Progress toward regional measles elimination-worldwide, 2000-2017. MMWR Morbidity and mortality weekly report.

[45] Gans H, Yasukawa L, Rinki M, DeHovitz R, Forghani B, Beeler J (2001). Immune responses to measles and mumps vaccination of infants at 6, 9, and 12 months. The Journal of infectious diseases.

[46] Griffin DE, Lin W-H, Pan C-H (2012). Measles virus, immune control, and persistence. FEMS Microbiology Reviews.

[47] Musa S, Topalovic B, Catic S, Smajlagic Z (2018). Assessment of vaccine effectiveness during measles outbreak in the Federation of Bosnia and Herzegovina, 2014-2015. Cent Eur J Public Health.

